# Targeting necroptosis in fibrosis

**DOI:** 10.1007/s11033-023-08857-9

**Published:** 2023-11-01

**Authors:** Emad H. M. Hassanein, Islam M. Ibrahim, Mostafa S. Abd El-Maksoud, Mostafa K. Abd El-Aziz, Esraa K. Abd-alhameed, Hanan S. Althagafy

**Affiliations:** 1 Department of Pharmacology and Toxicology, Faculty of Pharmacy, Al-Azhar University, Assiut, Egypt; 2 Graduated Student, Faculty of Pharmacy, Al-Azhar University, Assiut Branch, Assiut, 71524 Egypt; 3 Department of Pharmacology and Toxicology, Faculty of Pharmacy, Beni-Suef University, Beni-Suef, Egypt; 4 Department of Biochemistry, Faculty of Science, University of Jeddah, Jeddah, Saudi Arabia

**Keywords:** Necroptosis, Fibrosis, RIPK1, RIPK3, MLKL

## Abstract

Necroptosis, a type of programmed cell death that resembles necrosis, is now known to depend on a different molecular mechanism from apoptosis, according to several recent studies. Many efforts have reported the possible influence of necroptosis in human disorders and concluded the crucial role in the pathophysiology of various diseases, including liver diseases, renal injuries, cancers, and others. Fibrosis is the most common end-stage pathological cascade of several chronic inflammatory disorders. In this review, we explain the impact of necroptosis and fibrosis, for which necroptosis has been demonstrated to be a contributing factor. We also go over the inhibitors of necroptosis and how they have been applied to fibrosis models. This review helps to clarify the role of necroptosis in fibrosis and will encourage clinical efforts to target this pathway of programmed cell death.

**Graphical abstract:**

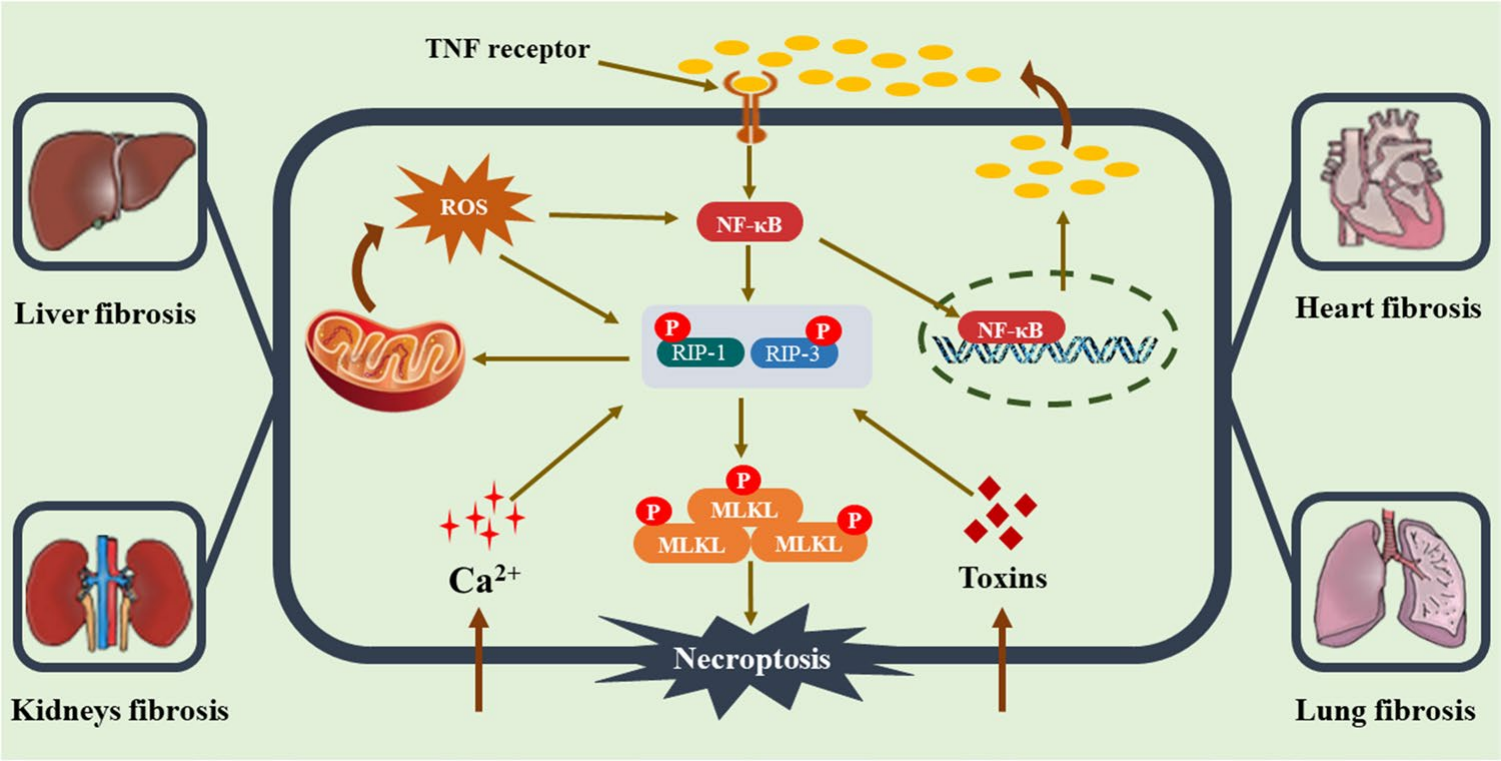

## Introduction

### Fibrosis overview

Up to 45% of deaths in the industrialised world are caused by fibrosis, which can damage any organ. Preclinical models and clinical experiments in a variety of organ systems have revealed that fibrosis is a highly dynamic process, contrary to long-held beliefs that it is relentlessly progressing and irreversible. A translational gap still exists between the identification of prospective anti-fibrotic targets and their conversion into efficient therapy despite tremendous advancements in our understanding of the pathobiology of fibrosis [1, 2].

**Fig. 1:**
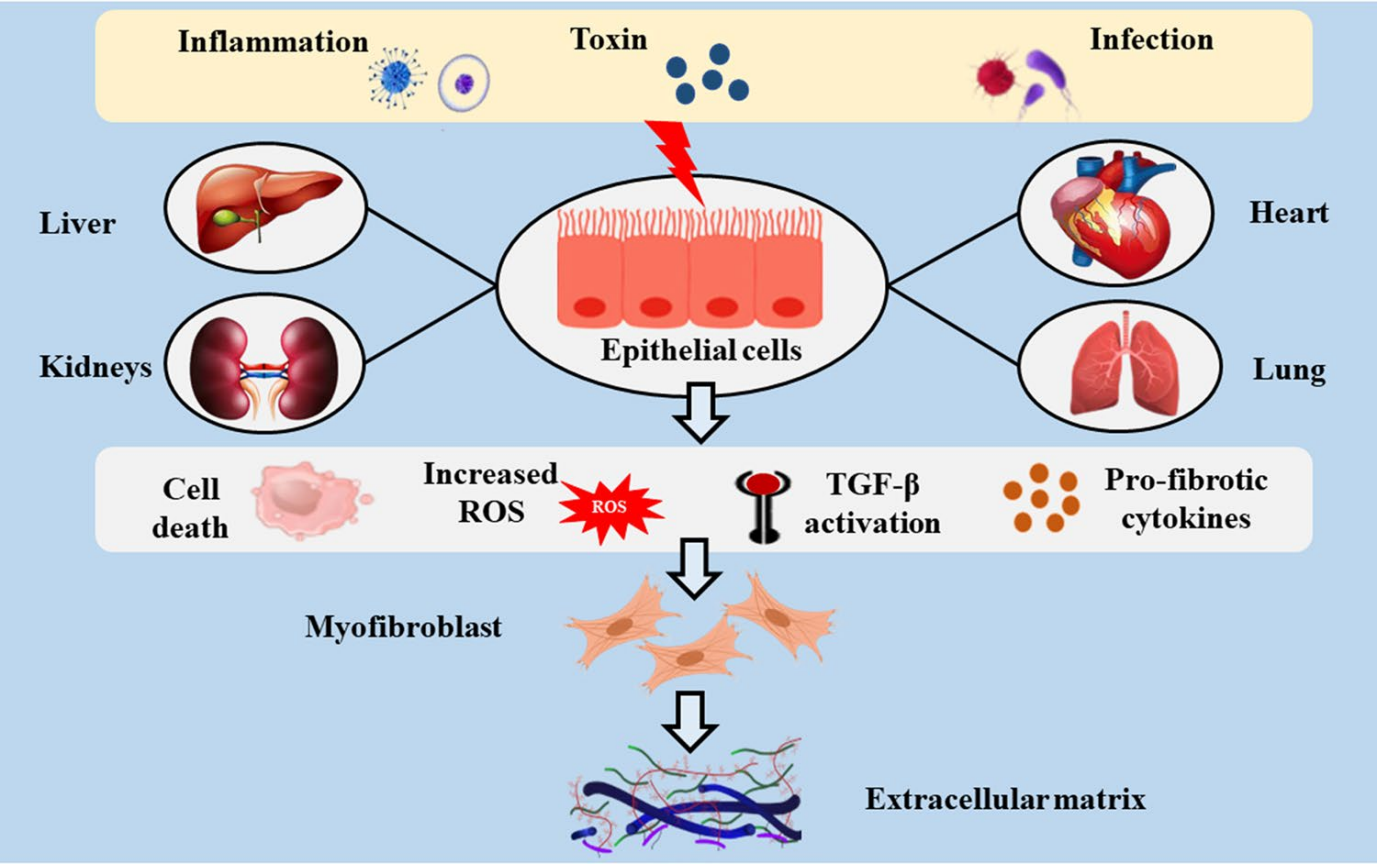
The main mechanism of fibrosis

Fibrosis is the most common end-stage pathological cascade of numerous chronic inflammatory disorders. Fibrosis is characterized by an overabundance of fibrous connective tissue (ECM components such as collagen and fibronectin) in the inflamed or destroyed tissue, which might lead to irreversible scar tissue formation, organ failure, and subsequently death [3–5]. Infections, autoimmune reactions, allergies, chemical assaults, radiation, and tissue damage contribute to chronic inflammation and fibrosis induction [2]. Fibrosis results after long-term exposure to various stimuli and involves inflammation. Despite their varied etiologies and clinical presentation, most chronic fibrotic disorders promote the release of growth factors and fibrogenic cytokines that severely damage normal anatomical structures [6, 7]. The recruitment of ECM-producing myofibroblasts is a hallmark common to all organ fibrosis [8, 9]. (As shown Fig. 1).

### Liver fibrosis

Liver fibrosis is an immediate and long-term reaction to liver damage [3]. The activation of HSCs, which have a role in myofibroblasts phenotyping, is a critical event in liver fibrosis [10, 11]. Active HSCs are proliferative, expressing alpha-smooth muscle actin (SMA), collagen type I and III secretion, and expression of matrix metalloproteinases (MMPs) [12–16]. The stimulation of HSCs is concluded in two key steps, initiation and perpetuation, followed by fibrosis [17]. Activating HSCs causes ECM buildup and chronic inflammation. Other ECM-producing cells, such as portal fibroblasts, myofibroblasts from bone marrow, and epithelial cells undergoing endothelial mesenchymal transition (EMT), also contribute to liver scarring [18–20]. Nevertheless, in the presence of transforming growth factor (TGF), oval cells may undergo EMT, enhancing the expression of HSC markers [21]. Moreover, throughout all stages, inflammatory cell recruitment is critical manifested by the presence of macrophages, which increased cytokine production, such as interleukin-13, causing liver fibrosis [22].

#### Lung fibrosis

Chronic damage to the alveolar epithelium may be caused by various causes, including smoking and viral infections [23]. Studies have shown that the immune system plays a significant role in the development of lung fibrosis. Interestingly, there is overwhelming evidence that innate and adaptive immunity plays a significant role in the etiology of pulmonary fibrosis. Idiopathic fibroblast proliferation and ECM modification are hallmarks of pulmonary fibrosis, a progressive and generally fatal lung disease [24, 25]. Innate and adaptive immune systems are linked by the pattern recognition receptors (PRRs) expressed on toll-like receptors (TLRs), which may be seen as master regulators of tissue structural and functional integrity [26].

#### Renal fibrosis

Chronic kidney disease (CKD) is a significant epidemiological clinical problem with a high prevalence and mortality rate. End-stage renal disease can be developed from CKD and result in serious complications [27]. Diabetic nephropathy, hypertension, and chronic interstitial glomerulonephritis are the most common causes of CKD. These diseases can cause structural and functional changes in the kidney. Chronic inflammation can cause renal fibrosis and is a major predisposing factor in CKD [28]. In addition, various cells, like macrophages, participate in renal fibrosis [29–31]. Renal fibrosis frequently leads to renal interstitial fibrosis with tubular atrophy and abnormal ECM deposition as the main pathological causes [32]. Renal fibrosis characterized by inflammatory cell infiltration, fibroblast activation, ECM component deposition, and microvascular thinning [33]. Many molecules and cells, such as angiotensin II (Ang II), are linked to the progression of renal fibrosis [34]. Animal models such as surgical, chemical, physical, genetic, and in vitro models are essential for understanding renal fibrosis biopathology and evaluating new therapies [35]. However, there are no available drugs for renal fibrosis. As a result, improving our understanding of renal fibrosis's cellular and molecular mechanisms is critical to eliminating renal fibrosis [36]. Alleviation of fibrosis alone is not sufficient to repair kidney function without restoring lost nephron tissue after damage. It is worth noting that encouraging endogenous tissue regeneration could be a promising treatment option for kidney disease [37].

#### Cardiac fibrosis

Cardiovascular diseases (CVDs) approximately cause 31% of all deaths worldwide [38], and cardiac fibrosis (CFs) impacts heart failure and end-stage remodeling of ECM. CFs differentiate into myofibroblasts (myoFbs) during cardiac injury [39]. MyoFbs show proliferative and invasive properties in response to disease or other stimuli. MyoFbs also remodel the interstitium by secreting MMPs that degrade ECM, increase collagen turnover, and enhance collagen net formation [40]. CFs, a scarring event in the cardiac muscle, occurs in nearly every type of heart disease, such as myocardial infarction (MI), diabetic cardiomyopathy, and aortic stenosis [41, 42]. ECM components' turnover is important in the fibrosis process, which is pathologically defined by increased deposition of type I and III collagens [43, 44].

### Necroptosis overview

There are numerous differences between apoptosis and necroptosis. Apoptotic cells preserve the viability of their cell membranes morphologically. In contrast, cells experiencing necroptosis demonstrate the breakdown of their cell membranes, a significant feature of necrosis. As a result, using traditional histologic methods, necroptotic cells are indistinguishable from necrotic ones despite the same stimuli [45]. Apoptosis and necroptosis have different intracellular signaling mechanisms that lead to their implementation. As caspases play an important role in apoptosis, receptor-interacting protein kinases (RIPKs) play a significant role in necroptosis. There is a prominent cross-talk between apoptosis and necroptosis [46, 47].

Necroptosis is a non-caspase-dependent cell death that has been involved in the pathogenic mechanisms of distinct diseases. It is an exciting area closely related to apoptosis. It is controlled by a number of genes that trigger cell death in a predictable and orderly manner. It shares normal necrosis features, such as loss of metabolic function and subcellular alterations, by activating specialized death signaling pathways [48, 49]. The first signaling molecule discovered in the necrosome was RIPK1 [50]. RIPK1 and RIPK3 interact with the receptor protein, which phosphorylates the mixed lineage kinase domain-like protein (MLKL) [51–53].

Necroptosis mainly regulates several signals, such as the caspase-8, nuclear factor-κB (NF-κB), and the mitogen-activated protein (MAP) kinase cascade. Many efforts have investigated the possible influence of necroptosis on human disorders. Interestingly, necroptosis plays a key role in the pathophysiology of various diseases, including hepatic diseases, renal injuries, human cancers, and others [54].

#### Necroptosis activation and signaling

Necroptosis signals have been thoroughly explored with the discovery of necrostatins as specialized inhibitors targeting RIPK1 [55–57]. Among the different models, the precious model is tumor necrosis factor (TNF)-α-induced necroptosis. After TNF-α binds to TNF receptor (TNFR)1, the adaptor molecules Fas-associated death domain (FADD) and TNF-receptor-associated death domain recruit RIPK1, which then binds RIPK3 to create the 'necrosome' complex [51, 52, 58, 59]. The oligomerization of RIPK3 and RIPK1 via the RIPK homotypic interaction motif (RHIM) domain causes autophosphorylation of RIPK3, which culminates in RIPK3 stimulation. Further early players, such as Toll/IL-1 receptor domain-containing adaptor-inducing IFN- (TRIF) and DAI, employ the RHIM domain to stimulate RIPK3, suggesting that the RHIM domain is involved in necroptosis. RIPK3 activation phosphorylates MLKL, which is responsible for necroptosis execution [53, 60]. When MLKL is phosphorylated, a molecular switch is set off that allows MLKL to travel to the plasma membrane and disrupt it [61, 62]. Moreover, IFNs cause necroptosis by overexpression of protein kinase R (PKR). This PKR interacts with RIPK1 and promotes the creation of the PKR necrosome, which consists of PKR, RIPK1, and RIPK3 [63]. Notably, IFNs are key in maintaining the RIPK1–RIPK3 complex activation [64].

**Fig. 2:**
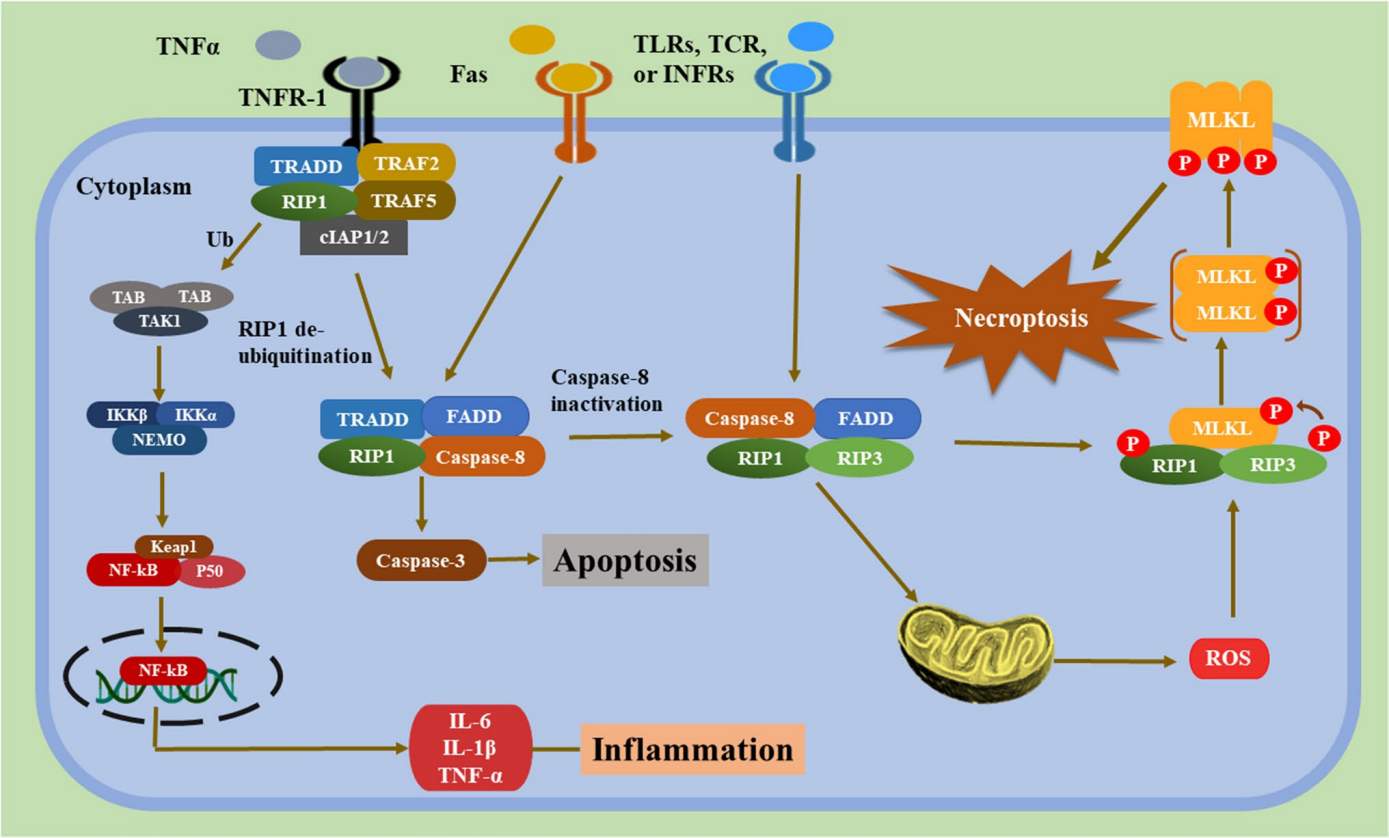
Molecular mechanisms of necroptosis

Necroptosis is divided into three types according to the causes that trigger it: TNF-α stimulates extrinsic necroptosis, reactive oxygen species (ROS) induce intrinsic necroptosis, and ischemia stimulates intrinsic necroptosis. TNF-α -mediated necroptosis is a kind of necroptosis in which TNF-α binds to a complementary receptor, creating a short-lived membrane signalling complex (complex I) [65, 66]. Consequently, TRAF2/3/5 and cIAPs are then recruited to Complex I [67]. During activation, cIAP1/2 and TRAF2/5 cause RIPK1 to be ubiquitinated, creating stable complex I and starting an alternate route that leads to cell survival via the NF-кB and MAPK signals [68]. (As shown in Fig. 2).

#### Necroptosis and inflammation

In contrast to apoptosis, cytokine production is a primary process leading to substantial inflammation following necroptosis. Necroinflammation, or the loss of plasma membrane integrity, which leads to the release of damage-associated molecular patterns (DAMPs) and the aggravation of tissue damage, is a unique characteristic of necroptosis [61]. After MLKL insertion, RIPK3 activates the inflammatory response mostly through the production of DAMP from cells. According to recent research, RIPK3 also triggers caspase-1 and caspase-11 by directly activating inflammasome in response to cellular stress or microbial infection [69, 70]. RIPK3 has been demonstrated in different investigations to increase cytokines release and inflammasome activation [71–73]. MLKL has been shown to be required for RIPK3-dependent inflammation [74]. However, it is uncertain how RIPK3 promotes NLPR3-mediated inflammasome production and whether or not MLKL is involved. According to certain theories, RIPK3 is a scaffold for complexes comprising RIPK1, FADD, and caspase-8 [70, 75, 76]. RIPK1 stimulates cytokine production independently of RIPK3 despite the presence of RIPK3. RIPK1 works as a scaffold in some models, particularly during TNF-mediated NF-κB and JNK activation, which leads to cytokine release [77, 78]. Following TLR4 activation, it was recently shown that RIPK1 is required to raise the circulating IL-1β, which is required to activate NF-κB, FOS, and ERK [79].

#### Necroptosis and oxidative stress

Oxidative stress can lead to necroptosis, which has been proven by several investigations [80–83]. Hydrogen peroxide induces necroptosis in RPE cells [84], and oxidative injury caused by paraquat produces necroptosis in cardiac muscles [85], indicating that oxidative stress plays a role in necroptosis. In mice, acetaminophen toxicity resulted in the formation of ROS and necroptosis [86]. Hyperoxia-induced oxidative injury resulted in necroptotic cell death in the pulmonary tissues of rats [87]. Researchers discovered that indicators of necroptosis are elevated in *Sod1*^*−/−*^ mice, which have a high level of oxidative stress [88, 89]. The age-related rise in oxidative stress is associated with an increase in necroptosis, based on an existing study indicating oxidative stress may trigger necroptosis mediated by a decrease in redox-sensitive pathway Nrf2 [90].

Some evidence demonstrated that ROS generation by mitochondrial respiratory complex I was critical for the necrotic response of L929 cells to TNF in the early 1990s. This was the first-time mitochondrial energy metabolism related to necrosis execution [91]. Cell death-related ultrastructural alterations in the mitochondria and endoplasmic reticulum (ER) are also mediated by mitochondrial ROS [91, 92]. RIPK3 action may connect TNFR1 signaling, mitochondrial bioenergetics, and ROS overproduction, even though ROS production is not required for all cases of TNF-induced necrosis [92]. TNF-induced necrotic cell death is additionally accelerated by the creation of non-mitochondrial ROS by the plasma membrane NADPH oxidase NOX1 (which is attracted by RIPK1) [93]. NOX1 is activated by TNF, and NOX1-induced ROS may induce or maintain the mitochondrial respiratory chain's further generation of ROS [94]. RNS, like ROS, are powerful oxidants that can begin or amplify lipid, protein, and peroxidation oxidation [95, 96]. Nitric oxide acts as a second messenger in a variety of signaling pathways at low intracellular concentrations. Nitric oxide is particularly detrimental when overproduced, and it produces RNS with different chemical and biological features [97]. Nitric oxide has recently been demonstrated to cause RIPK1- and RIPK3-mediated necroptosis [98].

## Role of necroptosis in the progression of organ fibrosis

### Liver fibrosis

Interestingly, necroptosis may play a critical role in different liver diseases. As explained previously, necroptosis is commonly seen in hepatocytes, and it is always accompanied by inflammation [99–101]. MLKL deficiency reduces glucose intolerance and hepatic insulin resistance [102]. By inhibiting hepatocyte autophagy, MLKL knockout protects against NASH induced by a high-fat, fructose, and cholesterol diet [103]. Targeting MLKL may be a successful strategy for treating liver fibrosis because MLKL-mediated signalling plays a significant role in liver damage and fibrosis [104]. Therefore, we addressed the concerns about necroptosis and its important role in liver inflammation and fibrosis.

Hepatic RIPK3 has been investigated as a factor in NAFLD severity in people and mice by Afonso et al. [105]. In this study, TNF-α promotes RIPK3-dependent oxidative stress through hepatocyte necroptosis. Hepatic RIPK3, MLKL, and TNF-α expression levels were increased in HFCD and MCD diet-fed animals. Strikingly, RIPK3 deficiency reduced MCD diet-induced liver fibrosis [105]. In line with this study, Majdi [106] and colleagues showed that the inhibition of RIPK1 improved NASH characteristics in HFD-fed mice and reversed steatosis through an MLKL-dependent mechanism that mainly affects mitochondrial respiration. They revealed that RIPA-56 inhibits RIPK1 to decrease liver damage, inflammation, fibrosis, and steatosis in animal models in either a curative or preventative manner. RIPA-56 or necrosulfonamide, a selective inhibitor of human MLKL, and the KO of MLKL in fat-loaded AML-12 murine hepatocytes were used. In steatotic hepatocytes, MLKL-KO activated mitochondrial respiration and increased β-oxidation. Additionally, in experimental NASH, RIPK3-KO animals showed higher activity of the liver mitochondrial respiratory chain complexes, which is consistent with lowered MLKL activation [106]. In the same context, Mohammed et al. [107] used a mouse model that produces spontaneous NAFLD/NASH and develops into fibrosis and HCC with age. MLKL and p-MLKL were markedly upregulated in the livers of Cu/Zn superoxide dismutase deficient (Sod1/ or Sod1KO) compared to WT mice. Similarly, RIPK3 and p-RIPK3 also increased. Also, NLRP3 inflammasome and transcript levels of proinflammatory cytokines and chemokines (TNF-α, IL-6, IL-1β, and Ccl2) linked with human NASH were upregulated in this study. In contrast, necrostatin-1 (Nec-1) treatment reversed these effects on Sod1KO mice [107]. In (2021), Afonso and co-authors explored the function of RIPK3 in controlling liver metabolism, damage, and inflammation. To hinder NASH, RIPK3 and its complex signalling have emerged as a new viable treatment. NASH patients' RIPK3 levels were elevated in both cohorts, and this was shown to be associated with both inflammation and fibrosis of the liver. As a result, RIPK3 impairment reduced CDAA-induced inflammation and fibrosis and inhibited tumorigenesis in mice at both 32 and 66 weeks of age. There were nodules and enhanced hepatocellular proliferation in WT mice fed a CDAA diet for six months, which were decreased in RIPK3^−/−^ animals [108].

Besides, the kinase activity of the RIPK1 protein, particularly in hematopoietic-derived macrophages, has been shown to participate in the pathogenesis of NASH, according to Xu et al. [109]. The NASH phenotype of hepatic steatosis, liver damage, fibrosis, and decreased hepatic cell death and inflammation in Rip1K45A/K45A mice was dramatically relieved compared to WT mice. RIPK1 kinase activation induced inflammasome activation, necrosis, and cell death in both bone marrow-derived macrophages and mice primary Kupffer cells following induction of kinase activation by lipotoxicity and in vitro saturated fatty acids (palmitic acid) [109].

Importantly, it has been observed that necroptosis is mediated by RIPK3 as well as neutrophil-driven inflammation of the alcoholic liver in patients with end-stage cirrhosis. Patients with alcoholic cirrhosis may have higher RIPK3 and MPO levels as a sign of a bad prognosis. The liver levels of MPO and RIPK3 were shown to be associated with a higher Ishak score [110]. A new study by Zhang et al. [111] identifies OGT as an important inhibitor of necroptosis in hepatocytes, and OGT-LKO mice may serve as an efficient spontaneous genetic model of liver fibrosis in humans. Hepatocyte necroptosis and the onset of hepatic fibrosis are prevented by O-GlcNAc alteration. Patients with alcoholic liver cirrhosis and mice with liver damage caused by ethanol both have lower levels of O-GlcNAc. Overt necroptosis and increased protein expression levels of RIPK3 and MLKL were seen in OGT-deficient liver cells [111].

It was discovered for the first time in human and rodent acute-on-chronic liver failure (ACLF) by Kondo et al. [112] that RIPK1-mediated cell death is critical, and it was also shown that inhibiting RIPK1 might be a new therapeutic method for preventing the progression of vulnerable individuals from ACLF. LPS was provided to bile-duct ligated rats, and galactosamine (CCL4/GalN) was delivered to carbon tetrachloride (CT)-induced fibrosis mice. Nec-1 therapy lowered the severity of ACLF by reducing liver, kidney, and brain damage, paralleled by reduced hepatic and renal cell deaths. A mouse model of ACLF induced by CCL4/GalN showed similar hepatoprotective effects with RIPA56 [112]. Non-ACLF patients at risk of developing ACLF may benefit from targeting RIPK1 as a therapeutic target, as shown by these findings. According to a recent study by Bai et al. [113], M2-like macrophages protect the liver by reducing necroptosis-S100A9-necroinflammation in ACLF [113].

Interestingly, melatonin (MLT) was found to reduce hepatic hydroxyproline content, TGF-β1, smooth muscle actin expression levels, and hepatocellular damage in chronic CCl4 administration. By impeding necroptosis-associated inflammatory signalling, MLT reduced these increases and prevented liver fibrosis by downregulating RIPK1 and MLKL. Serum high-mobility group box 1 (HMGB1) and IL-1β were both reduced by MLT, as was the interaction between HMGB1 receptors for advanced glycation end products and these two other CCl4-induced changes (RAGE) [114].

Another study conducted liver samples of human primary biliary cholangitis patients, coinciding with thioflavin T labelling, and indicating stimulation of necroptosis through elevated RIPK3 and p-MLKL levels. BDL resulted in apparent markers of necroptosis. In this study, RIPK3 impairment inhibited BDL-induced necroinflammation and oxidative stress reduced in RIPK3^−/−^ animals at 3 days following BDL. RIPK3 deficiency also related with increased hepatic expression of HO-1 indicating bile acid toxicity and buildup of iron in BDL mice. In PBC patients, necroptosis is initiated and facilitates hepatic necrosis in acute cholestasis produced by BDL. However, it may be necessary to use other strategies to prevent chronic liver disease development in necroptosis-targeted acute cholestasis [115].

Finally, Mohammed et al. [116] demonstrated that liver aging is accompanied with an increase in necroptosis, and necroptosis leads to chronic liver inflammation and fibrosis. Changes in necroptotic indicators in the liver were shown to correlate with changes in the expression of TNF-α, IL6, and IL1β, as well as the expression of markers of inflammation (TGF-β1, IFN-γ). Necroptosis markers and proinflammatory cytokines were enhanced in aged mice hepatocytes and liver macrophages compared to young mice. In contrast, Nec-1 decreased necroptosis, M1 macrophage markers, cell senescence, and the cytokines released in the livers of aged mice [116].

### Lung fibrosis

Recently, CoV-2 has been found to stimulate caspase-8, which causes apoptosis and inflammation of lung epithelial cells in response to SARS-CoV-2 infection. The virus-induced necroptosis mechanism releases the processed inflammatory cytokines. It has been shown that the SARS-CoV-2 virus causes apoptosis, necroptosis, and inflammatory activation in HFH4-hACE2 transgenic mice. In addition to necroptosis and apoptosis, a study of postmortem lung sections from COVID-19 patients found extensive inflammatory cell infiltration, necrotic cell debris, and pulmonary interstitial fibrosis. A treatment strategy for COVID-19 might benefit from these findings [117]. Latterly, Tao et al. [118] reported that macrophage-mediated necroptosis aids the course of silicosis by increasing pulmonary inflammation as well as fibrogenesis in their mouse model of the disease. Mice infected with silicosis show a substantial increase in necroptotic macrophage signaling in their lungs. In this silicosis model, the authors observed an increase in M1 macrophage infiltration and proinflammatory cytokines (TNF-α, IL-6). TGF-β1, fibrosis biomarkers α-SMA, and collagen I were likewise deregulated; however, Nec-1 was able to restore these effects [118].

In BLM-induced lung fibrosis and inflammation, Lee et al. (2018) found that necroptosis is linked to lung fibrosis via the release of damage-associated molecular patterns (DAMP). In idiopathic pulmonary fibrosis (IPF) lungs, RIPK3 expression was elevated, apoptosis, and necroptosis were mostly seen in alveolar epithelial cells (AECs). Knockout studies in AECs of Nec-1 and RIPK3 demonstrated that necroptosis is involved in BLM and hydrogen peroxide-induced cell death in necroptosis. AECs treated with BLM showed increased RIPK3, IL-1β, and HMGB1 expression levels. In RIPK3 mutant mice, Nec-1, as well as decreased HMGB1 and IL-1β levels, attenuated BLM-induced lung inflammation and fibrosis [119].

### Renal fibrosis

Several studies discussed the role of RIPK-mediated necroptosis in kidney disorders, including AKI and fibrosis [120]. In (2015), Zhu et al. found that necroptotic cell death, a higher level of RIPK1, and RIPK3 appeared eight weeks after subtotal nephrectomy surgery. Nec-1 treatment significantly improved renal functions. Nec-1 inhibited necroptosis as indicated by downregulating RIPK1, RIPK3, and MLKL [121]. Also, necroptosis has an important role in gentamicin-induced kidney injury progression; therefore, it is considered a potential target to alleviate AKI by inhibiting necroptosis. Upregulation of MLKL, RIPK3, p-MLKL was observed in gentamicin-treated mice and cultured renal tubule cells. In contrast, Nec-1 ameliorated gentamicin-induced necrosis and upregulated MLKL and RIPK3 in mice and in vitro cultured cells [122]. In cisplatin-induced nephropathy, disruption of TGF-β II receptor suppressed Smad2/3 activation and mitigated kidney injury. Smad2 evoked AKI by increasing apoptosis and inflammation, according to these findings. Smad2 knockout in TECs prevented renal function loss and reduced p53-mediated cell death, RIPK-evoked necroptosis, and NF-κB-induced inflammation. Lentivirus-mediated Smad2 knockdown mitigated kidney injury and inflammatory response while enhancing renal function [123].

In UUO-induced renal fibrosis, Dai et al. and co-workers showed the antifibrotic activity of fluorofenidone (AKF-PD) and Nec-1, which is mediated by the necroptosis suppression in TNF-α and Z-VAD stimulated HK-2 cells. They found that these agents ameliorate renal tubular damage and expression of IL-1β, TNF-α, and chemokines, as well as the deposition of collagen. Treatment with AKF-PD or Nec-1 protects renal tubular epithelial cells from necrosis and reduces the serum level of LDH-mediated by suppressing MLKL and RIPK3 phosphorylation [124]. In line with this study, Xiao et al. also reported that the levels of RIPK1/RIPK3/MLKL protein increased in the obstructed kidneys seven days after UUO. Interestingly, Nec-1 decreased TNF-α, IL-1β, and monocyte chemotactic protein-1 expression levels as well as TGF-β and α-SMA, indicating renal fibrosis suppression [125].

Zhu et al. [126] suggested that Ang-II exposure mediated necroptosis in renal tubular epithelial cells. They assess the necroptosis in the renal tubular cell in vivo by adding Nec-1 and in vitro with Ang-II and RIPK1/3 or MLKL inhibitors in HK-2 cells. Fas and FasL proteins have a key role in Ang-II-induced necroptosis, and FasL disruption reduced the percentage of necroptotic cells, implying that Fas and FasL are likely important signal regulators in Ang-II-induced necroptosis [126]. In addition, Zhu et al. speculated that necroptotic cell death might play a role in the loss of renal tubular cells in SNx rats. Effectively inhibiting necroptosis and apoptosis in CKD's early and intermediate phases improves renal function and tubular damage. Nec-1 and zVAD treatment significantly reduced necroptosis and apoptosis in SNx rats. Also, Nec-1 inhibited necroptosis and reduced the proportion of the TUNEL-positive cells [127].

### Cardiac fibrosis

Several investigations reported the potential impact of necroptosis heart diseases. Yue et al. [128] found that alliin could protect cardiomyocytes against necroptosis by maintaining cardiac function, decreasing myocardial lesions, and attenuating MI. In vitro and in vivo studies demonstrated that alliin prevented necroptosis while promoting autophagy. Alliin downregulated RIPK1, RIPK3, and TRAF2, while increasing Beclin-1 and LC-3 levels in a dose-dependent manner. In addition, alliin raises the level of PPAR-γ [128]. Zhang et al. have demonstrated that NaHS has anti-necrosis activity by reducing the expression of RIPK1 and RIPK3. NaHS also reduced the number of cardiac fibroblasts and the levels of α-SMA, proliferating cell nuclear antigen (PCNA), collagen I, and collagen III. In hypoxia-induced cardiac fibroblasts, NaHS boosted Sirtuin 3 (SIRT3) expression. Furthermore, the necroptosis inhibitory and antioxidant effects were decreased following SIRT3 siRNA transfection [129]. Also, Xu et al. reported the cardiac protective anti-inflammatory effects of irbesartan. The protective effects of irbesartan were related to inhibition of necroptosis as indicated by the downregulation of RIPK1, RIPK3, and MLKL levels [130]. Furthermore, Sharifi et al. and colleagues demonstrated that nesfatin-1 dose-dependently could exert a cardioprotective effect against MI/R by reducing oxidative stress and collagen deposition. Nesfatin-1 can suppress necroptosis by downregulating RIPK1, RIPK3, and MLKL [131].

Many studies discuss the cardioprotective effects of anti-necroptosis agents necrostatin-7 (Nec-7) and Nec-1 through inhibition of RIPK pathway. In 2022, Qiao et al. demonstrated that a modest dose of Nec-1 and GSK872 (GSK) inhibited RIPK1/RIPK3 in HGF-induced cardiac fibrosis. HGF activates the RIPK pathway, causing autophagic-related proteins such LC3-II, P62, and active-cathepsin D to be upregulated. The levels of RIPK3/p-RIPK3 and RIPK1/p-RIPK1 were both reduced when RIPK1/RIPK3 was inhibited. P62 forms a compound with both kinases and stimulates RIPK1 and RIPK3 binding. In a diabetic rat model, Nec-1 effectively reduced CF, decreased autophagic proteins, and enhanced heart function [132]. Furthermore, Wang et al. and co-workers found that Nec-1 ameliorates myocardial cell death by inhibiting fibrosis in rats with myocardial ischemia/late reperfusion. Nec-1 decreased creatine kinase and downregulated autophagy within 24 h after reperfusion. These findings imply that anti-necroptosis therapy may improve the therapeutic outcomes of ischemic heart patients [133]. In the same context, another study found that administration of Nec-7 before 1 h of left coronary arterial occlusion in the rat model reduced the scar length in the left ventricle on the 21st day after surgery. Nec-7 decreased N-terminal pro-brain natriuretic peptide, which improved left ventricular function [134]. In (2021) Fu et al. proved that necroptosis is a novel mechanism in AF pathogenesis in mice. Administration of CaCl2-Ach enhanced AF susceptibility and fibrosis in HFD model mediated by upregulating of RIPK1, RIPK3, MLKL, and CaMKs II. However, Nec-1 administration partially attenuated CaCl2-Ach or HFD-induced fibrosis, linking necroptosis to AF pathogenesis [135] (Table 1).

**Table 1:** summarizes the targeting of necroptosis by certain agents and the main effects

Agent	Organ fibrosis	Animal or cells	Main findings	References
Necrostatin-1	Liver	Mice	MLKL and p-MLKL were markedly upregulated in the livers of Cu/Zn superoxide dismutase deficient (Sod1/ or Sod1KO) compared to WT mice RIPK3 and p-RIPK3 also increased. Also, NLRP3 inflammasome and transcript levels of proinflammatory cytokines and chemokines (TNF-α, IL-6, IL-1β, and Ccl2) linked with human NASH were upregulated Nec-1 treatment reversed these effects on Sod1KO mice	[107]
RIPA-56 or necrosulfonamide,	Liver	Fat-loaded AML-12 murine hepatocytes and HFD-fed mice	RIPA-56 inhibits RIPK1 to decrease liver damage, inflammation, fibrosis, and steatosis in animal models in either a curative or preventative manner In steatotic hepatocytes, MLKL-KO activated mitochondrial respiration and increased β-oxidation In experimental NASH, RIPK3-KO animals showed higher activity of the liver mitochondrial respiratory chain complexes, which is consistent with lowered MLKL activation	[106]
Necrostatin-1	Liver	Mice	Nec-1 therapy lowered the severity of ACLF by reducing liver, kidney, and brain damage, paralleled by reduced hepatic and renal cell deaths. A mouse model of ACLF induced by CCL4/GalN showed similar hepatoprotective effects with RIPA56	[112]
Melatonin	Liver	Rats	MLT decreased hepatic hydroxyproline content, TGF-β1, smooth muscle actin expression levels By impeding necroptosis-associated inflammatory signalling, MLT reduced these increases and prevented liver fibrosis. MLT downregulated RIPK1, MLKL in liver tissue MLT decreased serum HMGB1 and IL-1β	[114]
Necrostatin-1	Liver	Mice	Nec-1 attenuated the increased levels of TNF-α, IL6, and IL1β, as well as the expression of markers of inflammation (TGF-β1, IFN-γ) in aged mice Nec-1 decreased necroptosis, M1 macrophage markers, cell senescence, and the cytokines released in the livers of aged mice	[116]
Necrostatin-1	Lung	Mice	Necroptosis is linked to lung fibrosis via the release of DAMP In IPF lungs, RIPK3 expression was elevated, apoptosis, and necroptosis were mostly seen in AECs Knockout studies in AECs of Nec-1 and RIPK3 demonstrated that necroptosis is involved in BLM and hydrogen peroxide-induced cell death in necroptosis AECs treated with BLM showed increased RIPK3, IL-1β, and HMGB1 expression levels In RIPK3 mutant mice, Nec-1, as well as decreased HMGB1 and IL-1β levels, attenuated BLM-induced lung inflammation and fibrosis	[119]
Necrostatin-1	Kidney	Rats	Nec-1 treatment significantly improved renal functions Nec-1 inhibited necroptosis as indicated by downregulating RIPK1, RIPK3, and MLKL	[121]
Necrostatin-1 and Fluorofenidone	Kidney	Mice	AKF-PD and Nec-1 decreased IL-1β, TNF-α, and chemokines, as well as the deposition of collagen AKF-PD and Nec-1 reduces the serum level of LDH-mediated by suppressing MLKL and RIPK3 phosphorylation	[124]
Necrostatin-1	Kidney	Rats	Nec-1 and zVAD treatment significantly reduced necroptosis and apoptosis Nec-1 inhibited necroptosis and reduced the proportion of the TUNEL-positive cells	[127]
Necrostatin-1	Kidney	Mice	Nec-1 downregulated RIPK1, RIPK3, and MLKL proteins Nec-1 decreased TNF-α, IL-1β, and monocyte chemotactic protein-1 expression levels as well as TGF-β and α-SMA	[125]
Alliin	Heart	Mice	Alliin prevented necroptosis while promoting autophagy. Alliin downregulated RIPK1, RIPK3, and TRAF2, while increasing Beclin-1 and LC-3 levels in a dose-dependent manner Alliin raises the level of PPAR-γ	[128]
Irbesartan	Heart	Rats	The protective effects of irbesartan were related to inhibition of necroptosis as indicated by the downregulation of RIPK1, RIPK3, and MLKL levels	[130]
Nesfatin-1	Heart	Rats	Nesfatin-1 suppress necroptosis by downregulating RIPK1, RIPK3, and MLKL	[131]
NaHS	Heart	Cardiac fibroblast	NaHS has anti-necrosis activity by reducing the expression of RIPK1 and RIPK3 NaHS reduced the number of cardiac fibroblasts and the levels of α-SMA, PCNA, collagen I, and collagen III NaHS boosted Sirtuin 3 (SIRT3) expression The necroptosis inhibitory and antioxidant effects were decreased following SIRT3 siRNA transfection	[129]
Necrostatin-1 and GSK872	Heart	Rats	Nec-1 and GSK872 inhibited RIPK1/RIPK3 in HGF-induced cardiac fibrosis In a diabetic rat model, Nec-1 effectively reduced CF, decreased autophagic proteins, and enhanced heart function	[132]
Necrostatin-1	Heart	Rats	Nec-1 ameliorates myocardial cell death by inhibiting fibrosis with myocardial ischemia/late reperfusion Nec-1 decreased CK and downregulated autophagy within 24 h after reperfusion	[133]
Necrostatin-1	Heart	Mice	Nec-1 administration partially attenuated CaCl2-Ach or HFD-induced fibrosis, linking necroptosis to AF pathogenesis mediated by downregulation of RIPK1, RIPK3, and MLKL	[135]

## Conclusions and future recommendations

Cell death can originate from pathological situations and is a natural process for replacing old cells. It is understood that cell death is a crucial component of both acute and chronic illnesses. Necroptosis is a mode of cell death that differs from apoptosis morphologically and biochemically. Necroptosis is mediated by intracellular signaling molecules, including RIPK1, RIPK3, MLKL, caspase-8, and others. Necroptosis is involved in many different diseases, according to preclinical research. Necroptosis has been shown to be a contributing factor in fibrosis. Consequently, liver, kidney, lung, and heart fibrosis have reportedly benefited from necroptosis inhibition. The inhibition of necroptosis motivates therapeutic efforts to pharmacologically target this mechanism of programmed cell death.

## Data Availability

Data sharing is not applicable to this article as no datasets were generated or analyzed during the current study.
